# Notification that new names of prokaryotes, new combinations and new taxonomic opinions have appeared in volume 76, part 6 of the IJSEM

**DOI:** 10.1099/ijsem.0.007258

**Published:** 2026-09-30

**Authors:** Aharon Oren, Markus Göker

**Affiliations:** 1 The Institute of Life Sciences, The Hebrew University of Jerusalem, The Edmond J. Safra Campus, 9190401 Jerusalem, Israel; 2 Leibniz Institute DSMZ – German Collection of Microorganisms and Cell Cultures, Inhoffenstrasse 7B, 38124 Braunschweig, Germany

This listing of names of prokaryotes published in a previous issue of the *International Journal of Systematic and Evolutionary Microbiology* (*IJSEM*) is provided as a service to microbiology to assist in the recognition of new names and new combinations. This procedure was proposed by the Judicial Commission [1]. The names given herein are listed according to the Rules of priority (i.e. time and date of publication of the articles on the journal’s platform and the order of valid publication of names in the original articles) [2].

**Table:** 

Order of article publication	Name/authors	Proposed as	Article no.	Page no.
1	*Metabacillus harenarius* Liang *et al*. 2026	sp. nov.	007185	6
2	*Legionella stuttgartensis* Rios Galicia *et al*. 2026	sp. nov.	007187	8
2	*Legionella nigrisilvae* Rios Galicia *et al*. 2026	sp. nov.	007187	8
3	*Nitrosomonas domestica* Zheng *et al*. 2026	sp. nov.	007183	6
3	*Nitrosomonas limosa* Zheng *et al*. 2026	sp. nov.	007183	6
4	*Pseudideonella* Lu and Chen 2026	gen. nov.	007181	8
4	*Pseudideonella sakaiensis* (Tanasupawat *et al*. 2016) Lu and Chen 2026	comb. nov. [basonym: *Ideonella sakaiensis* Tanasupawat *et al*. 2016]	007181	8
4	*Rhizobacter koreensis* (Chaudhary *et al*. 2022) Lu and Chen 2026	comb. nov. [basonym: *Schlegelella koreensis* Chaudhary *et al*. 2022]	007181	8
4	*Rhizobacter terrae* (Lu and Chen 2024) Lu and Chen 2026	comb. nov. [basonym: *Piscinibacter terrae* Lu and Chen 2024]	007181	8
5	*Salegentibacter profundi* Wang *et al*. 2026	sp. nov.	007188	8
5	*Salegentibacter haimaensis* Wang *et al*. 2026	sp. nov.	007188	8
6	*Acinetobacter uliginosus* Ran *et al*. 2026	sp. nov.	007192	7
6	*Acinetobacter halobius* Ran *et al*. 2026	sp. nov.	007192	7
7	*Clavibacter seminis* Arizala *et al*. 2026	sp. nov.	007184	21
7	*Clavibacter quasicaliforniensis* Arizala *et al*. 2026	sp. nov.	007184	21
8	*Latilactobacillus parasakei* Soto-Serrano *et al*. 2026	sp. nov.	007189	8
9	*Promicromonospora noduliphila* Alghannam *et al*. 2026	sp. nov.	007173	13
10	*Arcobacter pernae* Fang *et al*. 2026	sp. nov.	007197	8
10	*Arcobacter rhizosphaerae* Fang *et al*. 2026	sp. nov.	007197	8
11	*Fusobacterium abscessus* Kommedal *et al*. 2026	sp. nov.	007199	9
12	*Acinetobacter pengchengensis* Yang *et al*. 2026	sp. nov.	007174	9
13	*Sphingobium trunci* Budsabun *et al*. 2026	sp. nov.	007182	11
13	*Peribacillus folii* Budsabun *et al*. 2026	sp. nov.	007182	13
14	*Deinococcus superficialis* Zhang *et al*. 2026	sp. nov.	007193	11
15	*Nocardioides pymetrozini* Xu *et al*. 2026	sp. nov.	007186	7
16	*Allogeochorda* Kostovski and Oren 2026	gen. nov.	007208	2
16	*Allogeochorda subterranea* (Karnachuk *et al*. 2025) Kostovski and Oren 2026	comb. nov. [illegitimate basonym: *Geochorda subterranea* Karnachuk *et al*. 2025]	007208	2
16	*Allogeochordaceae* Kostovski and Oren 2026	fam. nov.	007208	2
16	*Allostigmatella* Kostovski and Oren 2026	gen. nov.	007208	2
16	*Allostigmatella ashevillensis* (Ahearne *et al*. 2025) Kostovski and Oren 2026	comb. nov. [illegitimate basonym: *Stigmatella ashevillensis* corrig. Ahearne *et al*. 2025]	007208	3
16	*Allostigmatella aurantiaca* (Berkeley and Curtis 1875) Kostovski and Oren 2026	comb. nov. [illegitimate basonym: *Stigmatella aurantiaca* Berkeley and Curtis 1875 (Approved Lists 1980)]	007208	3
16	*Allostigmatella erecta* (Schroeter 1886) Kostovski and Oren 2026	comb. nov. [basonym: ‘*Cystobacter erectus*’ Schroeter 1886]	007208	3
16	*Allostigmatella hybrida* (Reichenbach 2007) Kostovski and Oren 2026	comb. nov. [illegitimate basonym: *Stigmatella hybrida* Reichenbach 2007]	007208	3
16	*Allosphingorhabdus* Kostovski and Oren 2026	gen. nov.	007208	4
16	*Allosphingorhabdus lutea* (Baek *et al*. 2019) Kostovski and Oren 2026	comb. nov. [basonym: *Sphingorhabdus lutea* Baek *et al*. 2019]	007208	4
17	*Duganella kummerowiae* Huang *et al*. 2026	sp. nov.	007207	8
18	*Pseudarthrobacter cremeus* Baek *et al*. 2026	sp. nov.	007202	9
18	*Ideonella flava* Baek *et al*. 2026	sp. nov.	007202	9
19	*Chryseobacterium amylolyticum* Li *et al*. 2026	sp. nov.	007206	6
20	*Luteococcus struthionis* Miller *et al*. 2026	sp. nov.	007209	8
20	*Lacrimispora struthionigena* Miller *et al*. 2026	sp. nov.	007209	9
21	*Novosphingobium aeonii* Segura *et al*. 2026	sp. nov.	007220	10
22	*Pseudofrankia coriaricola* Dali *et al*. 2026	sp. nov.	007201	12
23	*Fictibacillus tiangongensis* Yuan *et al*. 2026	sp. nov.	007216	8
24	*Companilactobacillus miaonis* Wu *et al*. 2026	sp. nov.	007211	7
25	*Methylacidiphilum caldifontis* (Awala *et al*. 2023) Arahal *et al*. 2026	comb. nov. [illegitimate basonym: *Methylacidiphilum caldifontis* Awala *et al*. 2023]	007203	6
26	‘*Candidatus* Phytoplasma periplocae’ Siampour *et al*. 2026*	sp. nov.	007225	7
27	‘*Candidatus* Dedyshia’ Pieters *et al*. 2026*	gen. nov.	007213	9
27	‘*Candidatus* Dedyshia acidiphila’ Pieters *et al*. 2026*	sp. nov.	007213	10

*Name pro-validly published.

The following taxonomic opinions (i.e. the emendation of circumscriptions or the creation of synonyms) do not affect the status of a name under the International Code of Nomenclature of Prokaryotes. These taxonomic opinions can neither be considered as approved nor as disapproved by the International Committee on Systematics of Prokaryotes.

**Table:** 

Name/authors:	Article no.	Page no.
*Alkalispirochaeta alkalica* (Zhilina *et al*. 1996) Sravanthi *et al*. 2016 emend. Scranton *et al*. 2026	007205	4
*Alkalispirochaeta sphaeroplastigenens* (Vishnuvardhan Reddy *et al*. 2013) Sravanthi *et al*. 2016 pro synon. *Alkalispirochaeta alkalica* (Zhilina *et al*. 1996) Sravanthi *et al*. 2016	007205	4
*Desulfovibrio falkowii* Hamaguchi *et al*. 2025 emend. Kolsi *et al*. 2026	007212	5
*Kutzneria albida* (Furumai *et al*. 1968) Stackebrandt *et al*. 1994 pro synon. *Kutzneria viridogrisea* (Okuda *et al*. 1966) Stackebrandt *et al*. 1994	007200	4
*Kutzneria viridogrisea* (Okuda *et al*. 1966) Stackebrandt *et al*. 1994 emend. Dean *et al*. 2026	007200	4
*Latilactobacillus* Zheng *et al*. 2020 emend. Soto-Serrano *et al*. 2026	007189	8
*Rhizobacter* Goto and Kuwata 1988 emend. Lu and Chen 2026	007181	8
*Streptomyces atacamensis* Santhanam *et al*. 2012 pro synon. *Streptomyces radiopugnans* Mao *et al*. 2007	007194	6
*Streptomyces chitinivorans* Ray *et al*. 2016 pro synon. *Streptomyces fenghuangensis* Zhu *et al*. 2011	007194	5
*Streptomyces fenghuangensis* Zhu *et al*. 2011 emend. Komaki 2026	007194	8
*Streptomyces mangrovi* Wang *et al*. 2015 emend. Komaki 2026	007194	9
*Streptomyces pini* Madhaiyan *et al*. 2016 pro synon. *Streptomyces mangrovi* Wang *et al*. 2015	007194	6
*Streptomyces radiopugnans* Mao *et al*. 2007 emend. Komaki 2026	007194	9
